# A case of successful pembrolizumab monotherapy in a patient with advanced lung adenocarcinoma: Use of multiple biomarkers in combination for clinical practice

**DOI:** 10.1515/med-2021-0404

**Published:** 2021-12-15

**Authors:** Hanfei Guo, Lei Qian, Xiao Chen, Yuguang Zhao, Wei Song, Yanjie Guan, Jiuwei Cui

**Affiliations:** Cancer Center, The First Hospital of Jilin University, Changchun 130021, China

**Keywords:** immune checkpoint inhibitors, prognostic marker, hyperprogressive disease

## Abstract

Clinical treatment is challenging for elderly patients with lung cancer who cannot tolerate chemotherapy, do not have cancer driver genes, and have low expression of PD-L1. Since these patients are usually excluded from clinical studies, evidence-based medicine supporting the use of immunotherapy is lacking. Considering the potentially limited clinical benefits and high associated risk of hyperprogressive disease, determining an appropriate treatment is an urgent clinical challenge. We report a 71 year-old male patient diagnosed with advanced lung adenocarcinoma lacking key driving genes (*EGFR*, *ALK*, and *ROS-1*), and low expression of PD-L1 on tumor cells (10–15%). The tumor tissue showed a low level of microsatellite instability, low tumor mutational burden, and no DNA mismatch repair deficiency on whole-exome sequencing (WES). However, a high blood tumor mutational burden was detected. After considering the biomarkers of therapeutic effect and ruling out the risk of hyperprogressive disease, pembrolizumab 200 mg was administered every 3 weeks for a year (17 cycles). The disease remained stable for >39 months, and adverse effects were mild and well-tolerated. Therefore, a comprehensive biomarker evaluation, especially in elderly patients lacking driving genes, is essential. Liquid biopsy technology and WES may be useful for overcoming the limitations of tissue biopsy.

## 1 Introduction

Immune checkpoint inhibitors (ICIs) can be used to induce a durable anti-tumor immune response by overcoming immune tolerance and enhancing the activation of anti-tumor T cells [1]. The development of ICIs has prompted changes in cancer treatment strategies and gradually improved the efficacy of lung cancer treatment [2,3,4,5,6].

However, about half of the patients with non-small cell lung cancer (NSCLC) are aged >65 years [7], which the World Health Organization defines as elderly. Elderly patients often are in poor condition and are at risk for many complications; therefore, these patients have difficulty tolerating chemotherapy and radiotherapy [8]. Although immunotherapy is associated with relatively fewer side effects, there are certain negative treatment responses, such as hyperprogressive disease (HPD) and pseudo-progression, which are reported to be more common in elderly patients [9,10]. Considering the differences in the mechanisms and toxicity of immunotherapy and chemotherapy for the elderly population, more individualized attention is needed when choosing treatment regimens.

Herein, we report a case of metastatic lung adenocarcinoma in an elderly patient. At the time of diagnosis, the patient was negative for all the key tumor driving genes, and the PD-L1 expression rate in the tumor tissue was 10–15%. As a result, he did not meet the criteria for targeted therapy and immunotherapy. The patient underwent whole-exome sequencing (WES), and through a comprehensive analysis of microsatellite instability (MSI), mismatch repair (MMR), tumor mutational burden (TMB), and other indicators (including risk factors for HPD), we determined that the patient could potentially benefit from ICI treatment. The patient received pembrolizumab monotherapy and achieved stable disease long-term without obvious immune-related toxicity. The application of predictive markers and genome-wide exome sequencing for guiding therapy is further discussed.

## 2 Case presentation

**Figure 1:**
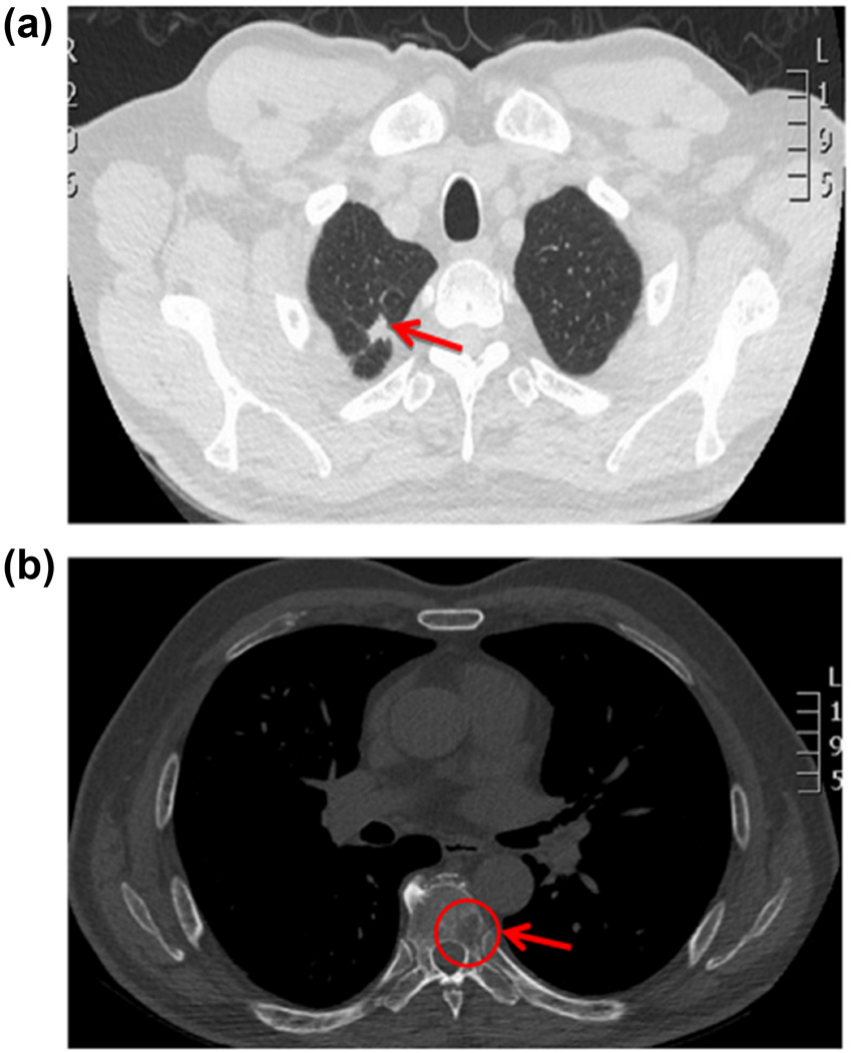
Imaging of the lung before diagnosis. (a) Consolidation of the lesion in the right upper lobe (May 2017). (b) Significantly damaged seventh thoracic vertebral bone (May 2017).

In May 2017, a 71 year-old male presented with multiple lung nodules on computed tomography (CT) during a routine physical examination (Figure 1a and b). Further PET-CT examination indicated right-sided lung cancer with a maximum diameter of 1.0 cm, hilar/mediastinal lymph node metastasis, and multiple bone metastases. Pathological examination revealed a moderately differentiated lung adenocarcinoma. The patient was staged as cT4N2M1b (stage IV) according to the seventh edition of the American Joint Commission on Cancer [11]. He had a history of hypertension, type 2 diabetes, and coronary atherosclerotic heart disease; had stable blood pressure and good blood sugar control; and an Eastern Cooperative Oncology Group (ECOG) physical status score of 1 point.

The immunohistochemistry results from the primary lung lesion were as follows: ALK-VentanaD5F3 (−), ALK-Neg (−), BRAF-V600E (−), Her-2 (−), ROS1 (2+), C-MET (2+), and EGFR (3+). Gene test results were negative for *EGFR*, *ALK*, and *ROS-1*; therefore, this patient had no alternative options for targeted therapy. The PD-L1 expression level of the tumor by immunohistochemical staining (Daco 22C3 antibody) was 10% positive in the primary lung lesions and 15% positive in the bone metastases. According to the 2017 guidelines, the patient did not meet the criteria for pembrolizumab monotherapy for NSCLC (tumor proportion score >50%) [3,12]; therefore, a combination of pembrolizumab and chemotherapy was recommended (approved in May 2017, regardless of PD-L1 expression status, KEYNOTE-021 trial) [13]. However, the patient refused chemotherapy treatment.

Since determining prognosis based on the expression of PD-L1 alone is difficult, we comprehensively analyzed other markers that could be used to predict the curative effect of ICI. WES of the blood and tissue specimens was performed on July 4, 2017. The TMB, also known as the mutation load, was reported to be 49 mutations/Mb in the primary lung tumor tissue. Blood TMB (bTMB), a novel summative measure of circulating tumor DNA (ctDNA) genomic alterations in the peripheral blood, was also conducted and was found to be high in our patient (408 mutations/Mb in the blood sample). The MSI of the lung cancer tissue was low (MSI-L) on exome sequencing, and among the five microsatellite detection sites (NR-21, NR-24, BAT-25, BAT-26, and MONO-27), only BAT-25 was unstable. On exon sequencing of the lung cancer tissue, the missing copy number of MSH6 was 1.35, suggesting MMR gene damage. Small insertions and deletions (indels) can generate a new open reading frame, producing a new immunogen that is more immunogenic than single-nucleotide polymorphisms, making it easier to activate the immune system using immunomodulatory blocking agents. The number of indels and the indel/TMB ratio are positively correlated with the efficacy of immunotherapy [14]. For this patient, the number of indels was 63, and the indels/TMB ratio was 15.4%. In addition, genes that have been reported to lead to HPD were all negative according to the WES results, so we considered that the patient was less likely to develop HPD (Table 1). In summary, we determined that this patient could potentially benefit from pembrolizumab monotherapy. After this comprehensive evaluation and a discussion of the personal wishes of the patient, we administered pembrolizumab monotherapy at a dose of 2 mg/kg every 3 weeks for 1 year (17 cycles).

**Table 1:** Summary of the patient’s main tissue and blood specimen analysis results

	Sample type		
	Primary lung lesion	Bone metastases	Blood
IHC staining results	ALK-VentanaD5F3 (−), ALK-Neg (−), BRAF-V600E (−), Her-2 (−), ROS1 (2+), C-MET (2+), EGFR (3+)	Ki-67 (+) (<5%), TTF1 (+), NapsinA (+), EGFR (+)	
PD-L1-positive rate	10% in tumor and 70% in tumor-infiltrating inflammatory cells	15% in tumor and 20% in tumor-infiltrating inflammatory cells	
Target drug-related gene sequencing	EGFR (−), KRAS (−), NRAS (−), BRAF (−), ALK (−), ROS-1 (−), RET (−)		
MMR gene damage	MSH6 missing copy number: 1.35	MLH1 (20%), MSH2 (5%), MSH6 (5%)	
TMB	49 mutations/Mb		408 mutations/Mb
MSI	0.06%		0.57%
Copy number variation (CNV) of chromosome			4.3%

**Figure 2:**
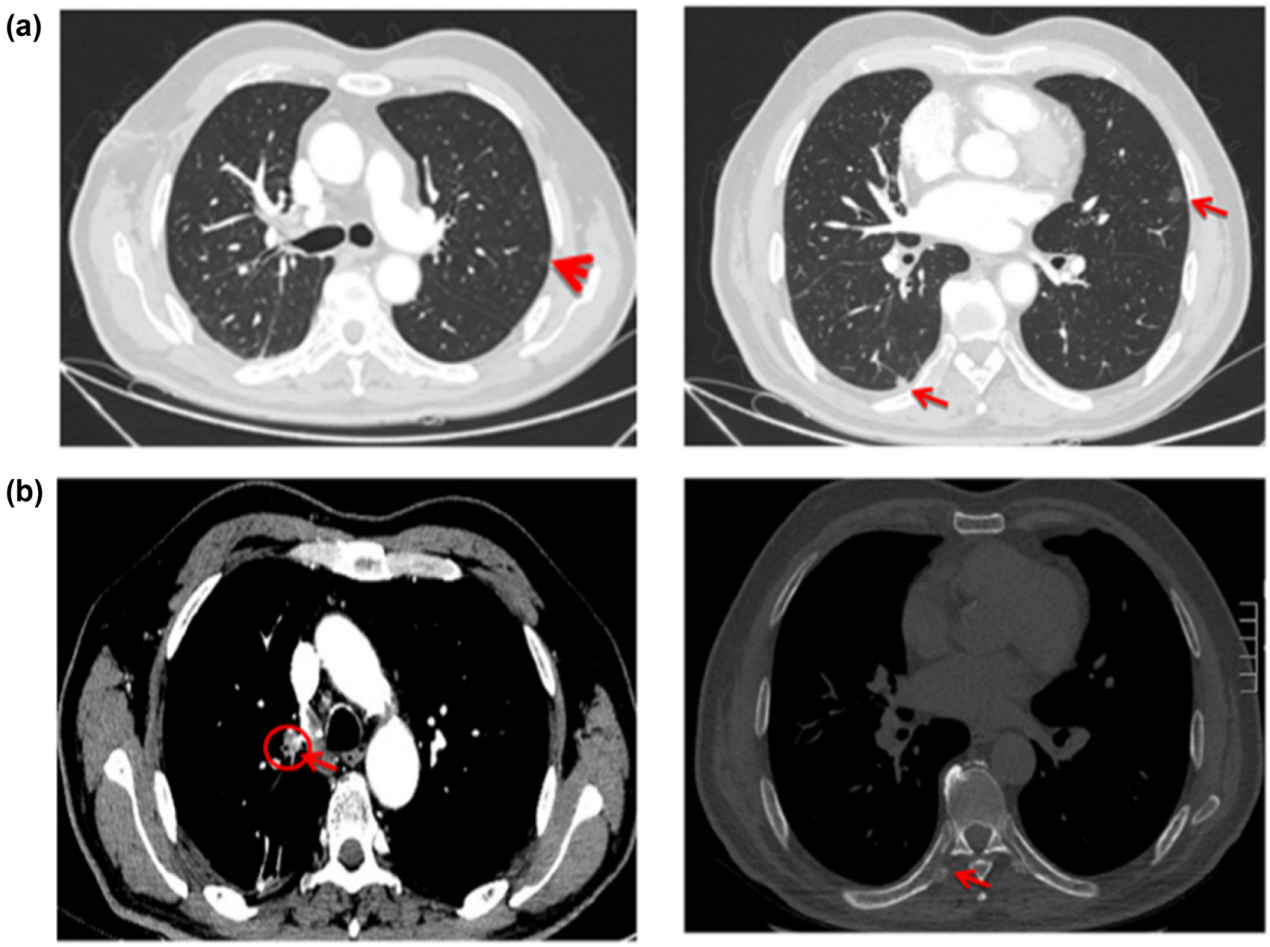
(a and b) Baseline CT of the pretreatment primary tumor lesions (July 17, 2017).

**Figure 3:**
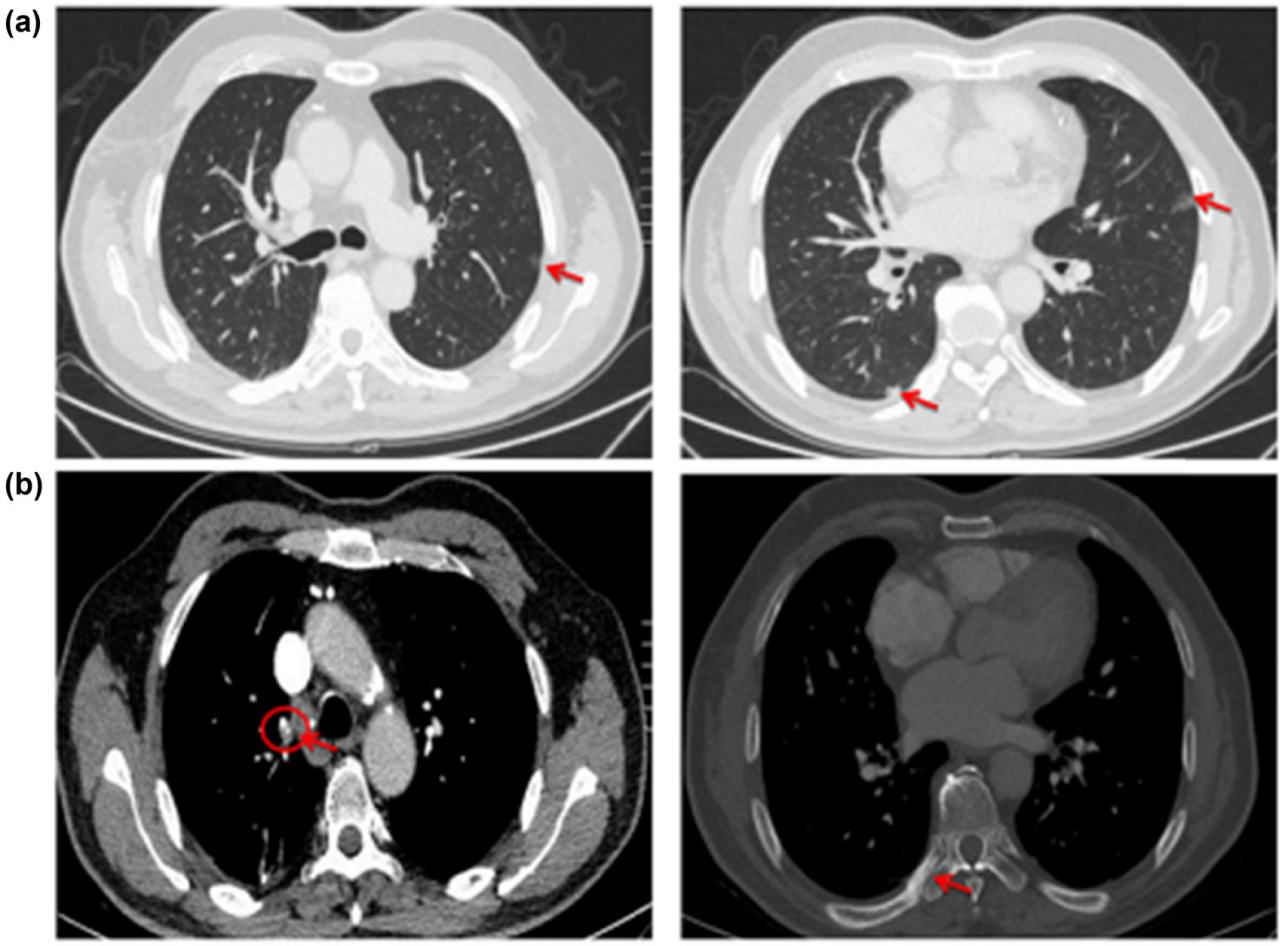
(a and b) Changes to the major tumor lesions after three courses of treatment (October 9, 2017). While there are no significant changes to the bilateral pulmonary nodules, the mediastinal lymph nodes are slightly smaller and destruction and repair of the rib bones are evident.

**Figure 4:**
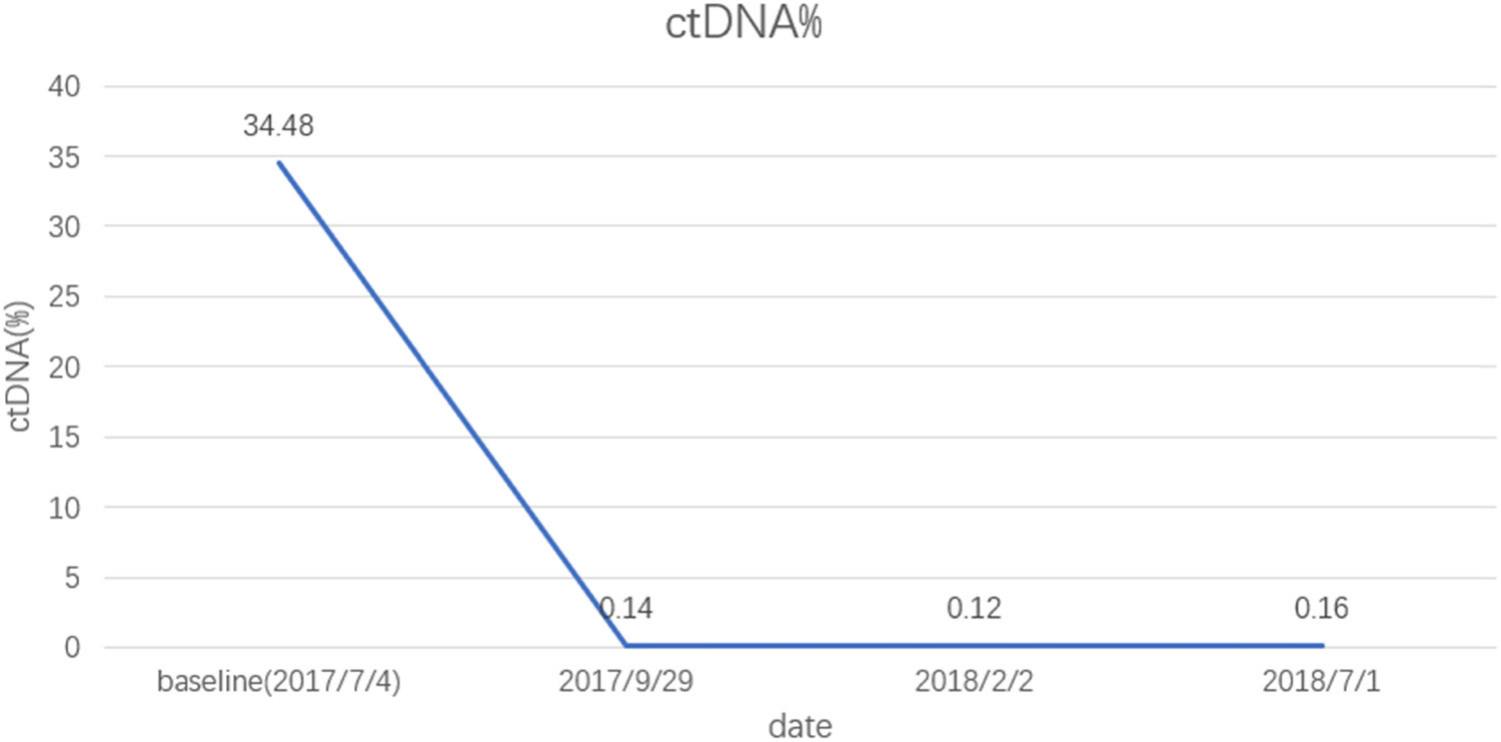
Variation of ctDNA abundance in the peripheral blood.

For the first evaluation of efficacy, which was conducted after three treatment courses (October 9, 2017) (Figures 2a and b and 3a and b), the patient was found to have stable disease according to the RECIST criteria. The ctDNA results provided additional confirmation of treatment efficacy. At baseline before the treatment (July 4, 2017), the first detection of ctDNA abundance (ctDNA abundance = ctDNA in peripheral blood/total free DNA in peripheral blood) was 34.48%, which subsequently decreased rapidly and stabilized below 1% (Figure 4).

During the 14th cycle of treatment (April 2018), the patient developed oral ulcers and dry mouth and was diagnosed with a fungal infection. The oral ulcers resolved after 2 weeks of oral nystatin and other oral antifungal medications. Additionally, the patient’s hormone levels showed hyperglycemia and subclinical hypothyroidism (Grade 1) in May 2018. The patient reported no obvious discomfort and received thyroid hormone replacement therapy (Euthyrox) and continued insulin to control blood sugar. After completing 17 courses of immunotherapy, the patient discontinued treatment. The patient’s blood glucose and thyroid hormone levels returned to normal within 2 months of discontinuing immunotherapy.

A total of 17 courses of pembrolizumab had been administered by July 2018, when the patient decided to discontinue ICI treatment. By September 2020, the patient had survived >39 months progression-free and continued to have stable disease.


**Ethics approval and consent to participate:** The patient agreed that the doctors could use and publish his disease related article with personal information deleted.
**Consent to publish:** See in the supplementary material.

## 3 Discussion

ICI therapy has successfully improved the treatment options for NSCLC. However, while it has significantly improved the prognosis in patients with lung cancer, three major challenges with immune monotherapy must be considered, namely, prediction of efficacy, HPD, and the early evaluation of efficacy. Elderly patients aged >65 years are especially vulnerable since they often have poor tolerance to chemotherapy and are prone to developing HPD after immunotherapy. Currently, no single predictive markers are known that can manage these challenges. The comprehensive consideration of PD-L1 and MMR in tumor tissue samples and of WES and ctDNA in tissue and blood samples could provide patients who are not suitable for immunotherapy (according to the NCCN guidelines) with long-term survival benefits.

Currently, predicting efficacy is a major challenge in immunotherapy. While there are many biomarkers that can be used to predict the efficacy of immunotherapy, they all have limitations. Additionally, the heterogeneity of tumors and spatiotemporal differences in tissue sampling can also affect the predictive capacity of biomarkers. Measuring the protein levels of PD-L1 has been the first candidate biomarker for anti-PD-1/PD-L1 therapy. Since PD-L1-positive tumors respond better to PD-1 inhibitor treatment (KEYNOTE-010 and KEYNOTE-024) [3,15], the National Comprehensive Cancer Network guidelines recommend pembrolizumab monotherapy as first-line treatment for NSCLC tumors with high expression of PD-L1 (tumor proportion score ≥50%) [16]. However, patients with negative/low expression of PD-L1 are still likely to benefit from ICI treatment [17]. Mismatch repair defects (dMMR)/high MSI (MSI-H) can cause various somatic mutations and produce new antigens [18], which are more responsive to immunotherapy [19,20,21]. The FDA has approved pembrolizumab for the treatment of all types of solid tumors with MSI-H or dMMR [22]. Significant clinical benefits have also been shown in patients with high TMB [23]. The correlations of TMB and PD-L1 were low [24]. In combination, these biomarkers may be useful for identifying the patients most likely to respond to anti-PD-1 therapies. In this report, the TMB was low, with 49 mutations/Mb in the lung issue and 408 mutations/Mb in the peripheral blood tissue. Additionally, while no dMMR was found in the bone metastases, full exon sequencing of peripheral blood showed significant MMR injury. A possible explanation may be the low tumor purity (30–45%) of clinical specimens obtained by fine needle puncture or bronchoscopy tissue [25]. Due to tumor heterogeneity, the circulating tumor cells may better reflect the tumor mutations overall compared to the tissue of the primary tumor. Moreover, obtaining adequate tumor tissue samples from patients with advanced disease can be challenging, and WES of peripheral blood provides an effective alternative method for predicting the patients’ prognoses. To date, a single marker that can specifically and accurately predict the efficacy of ICI has not been discovered; therefore, comprehensively analyzing various markers and assessing the risks and benefits for the individual patient is necessary.

Two other potential challenges to clinical treatment for this case were HPD and adverse effects, which are common in elderly patients. The incidence of HPD is approximately 5–10% and is more common (19%) in elderly patients (aged ≥65 years) [9,26]. In this patient, previously reported HPD-related genes such as *CCND1, CDK4, CDK6, EGFR, FGF19, FGF3, FGF4, DNMT3A, MDM2,* and *MDM4* were all negative on WES using tissues from both the lung and bone metastases. Consistent with this pretreatment assessment, HPD was not observed in this patient throughout the study period. The first evidence of adverse effects (oral ulcers and dry mouth) occurred more than a year after immunotherapy and disappeared 2 months after it was discontinued. The overall incidence of hypothyroidism during ICI treatment is 6.6% and usually occurs within the first year of treatment [27]. Thyroid dysfunction associated with ICIs may be permanent or require long-term thyroid hormone replacement therapy [28]. There is no consensus regarding the risk factors for ICI-related thyroid dysfunction; however, the occurrence of adverse reactions in the immune system was associated with better treatment response and survival benefits [29]. Moreover, with appropriate hormone replacement therapy, most patients have no clinical symptoms associated with thyroid dysfunction.

Due to the scavenging property of macrophages, the level of ctDNA in healthy individuals remains low, while the level in patients with tumors increases because of tumor cell necrosis and dissemination [30]. Since ctDNA can reflect dynamic changes at the molecular level, ctDNA clearance during treatment can be used as a predictor and prognostic marker for a variety of treatments [31]. At the patient’s first evaluation of treatment efficacy, the CT results showed a slight increase in tumor size (the total length and diameter of the target lesion increased by 10%), while the ctDNA was in a state of decline. The patient achieved long-term clinical benefits (progression-free survival >39 months), so we determined that the immunotherapy was effective. In the event that a patient has a dissociated response to immunotherapy (i.e., the volume of some tumors increases while that of others decreases after treatment), the criteria used to evaluate the curative effect of treatment remain weak [32]. One study found that the incidence of a dissociated response to anti-PD-1/PD-L1 treatment in patients with NSCLC was 7.5%; however, no relevant predictors of this type of response were identified [26]. Since radiographic imaging cannot be used to determine clonal evolution at the molecular level, ctDNA provides important information regarding the effectiveness of immunotherapy.

## 4 Summary

In conclusion, this case illustrates the successful use of pembrolizumab as a first-line treatment for a 71 year-old patient with lung adenocarcinoma. Many previous studies have shown that immunotherapy is equally safe and effective in elderly patients aged 65–75 years as it is in young patients [33,34]. For patients aged 75 to 80 years, however, the benefits of immunotherapy are reduced, potentially since more of these patients are in poor general condition (ECOG PS 2). Therefore, the presence of comorbidities and the general condition of patients aged 75–80 years may be important factors to consider when determining the potential benefits of immunotherapy [35,36]. Considering the need to balance survival benefits with quality of life and acknowledging that elderly patients show poor tolerance to radiotherapy and chemotherapy, older patients without driving genes should be offered immune therapies when possible.

Consequently, the comprehensive evaluation of different biomarkers to identify more effective treatment options, especially in elderly patients with negative driving genes, is essential. In the future, WES and/or the indel/TMB ratio may become powerful biomarkers of immunotherapy after more large-sample clinical studies are conducted that provide comprehensive treatment results. Additionally, future studies on the mechanisms of immunotherapy in elderly patients and prognosis biomarkers are needed.

## Abbreviations

HPD: hyperprogressive disease
MSI-L: microsatellite instability
TMB: tumor mutational burden
MMR: mismatch repair
WES: whole-exome sequencing
bTMB: blood TMB
NSCLC: non-small cell lung cancer
PP: pseudo-progression
CT: computed tomography
TPS: tumor proportion score
SNP: single-nucleotide polymorphism
SD: stable disease
ctDNA: circulating tumor DNA
NCCN: National Comprehensive Cancer Network
dMMR: mismatch repair defects
MSI-H: high microsatellite instability
